# Exploring the roles of cytochrome P450 enzymes and their inhibitors in cancers and non-neoplastic human diseases

**DOI:** 10.1007/s12272-025-01581-x

**Published:** 2025-11-18

**Authors:** Hyein Lee, Yeo-Jung Kwon, Young-Jin Chun

**Affiliations:** College of Pharmacy and Center for Metareceptome Research, Chung-Ang University, 84 Heukseok-Ro, Dongjak-Gu, Seoul, 06974 Republic of Korea

**Keywords:** Cytochrome P450 (CYP), Drug metabolism, Cancer, Inhibitor, Drug–drug interaction (DDI)

## Abstract

Cytochrome P450 (CYP) enzymes are crucial for metabolizing various compounds, including therapeutic drugs. Metabolites generated through CYP-mediated pathways have been increasingly recognized as key contributors to the pathogenesis and progression of diverse diseases, particularly cancer. Consequently, ongoing research is examining Food and Drug Administration-approved drugs as potential inhibitors of specific CYP isoforms and characterizing their underlying mechanisms of action. These studies are essential for clarifying how approved drugs alter the metabolic pathways of co-administered agents, thereby influencing therapeutic efficacy and safety outcomes. CYP inhibitors significantly alter substrate metabolism, thereby increasing the risk of drug–drug interactions (DDIs). These interactions pose crucial challenges in clinical practice, necessitating careful evaluation when co-administering medications with similar metabolic pathways. Therefore, this review aims to examine the complex interplay among CYP inhibitors, their substrates, and DDIs in both cancers and non-neoplastic diseases, including allergies, depression, and stroke. The review seeks to minimize adverse outcomes and enhance therapeutic effectiveness by offering a comprehensive understanding of CYP inhibitors.

## Introduction

Various types of enzymes orchestrate a broad range of metabolic processes in organisms, including humans. Enzyme inhibitors bind enzyme active sites to modulate their catalytic activity, offering potential treatments for diverse diseases (Copeland et al. 2007). The clinical use of numerous enzyme inhibitors underscores the importance of enzymes as viable drug targets (Copeland 2005). Several key enzymes involved in cellular and drug metabolism have emerged as promising therapeutic targets for diseases with limited effective treatment options (Robertson 2005; Orhan 2019; Zhao et al. 2021).

**Fig. 1:**
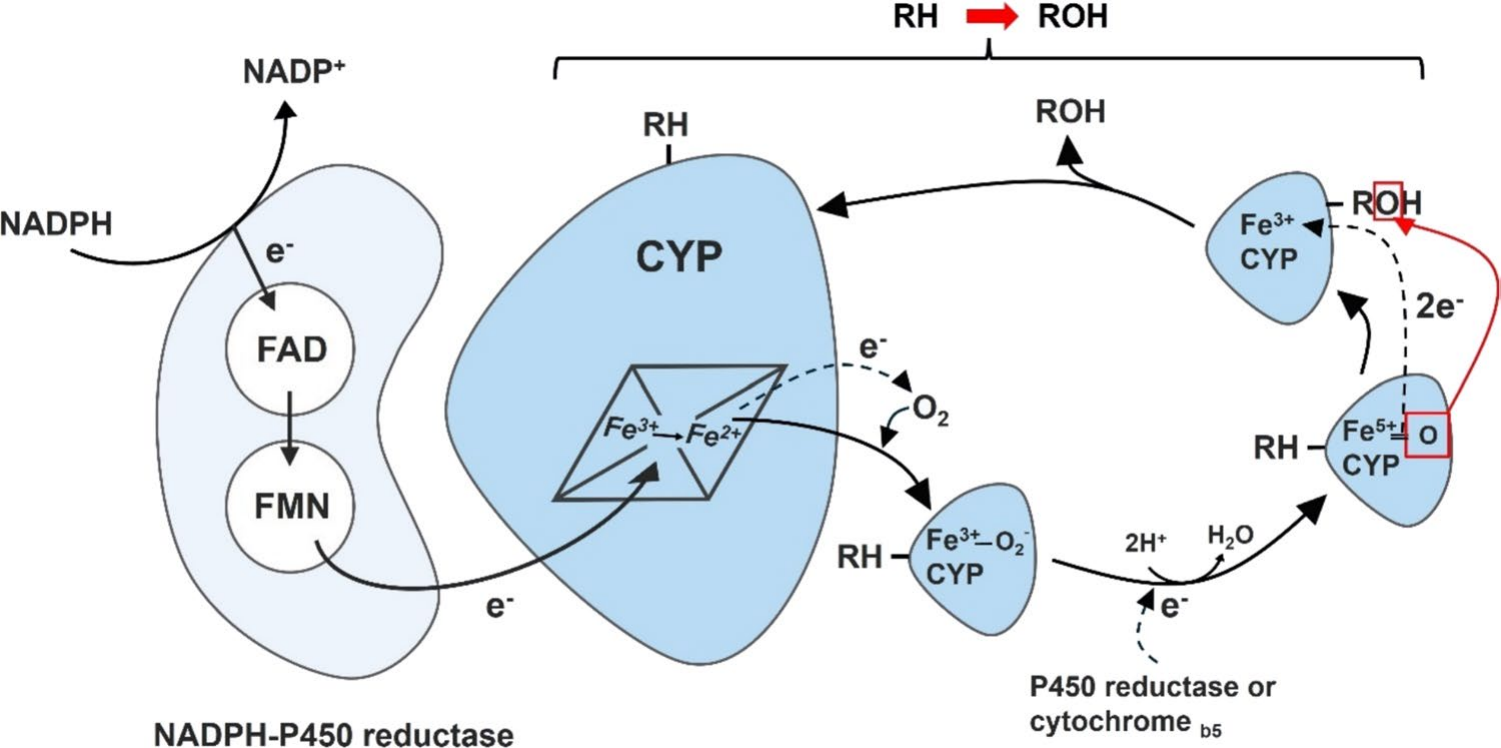
Role of human CYP enzymes and NADPH–P450 reductase in oxidative drug metabolism. CYPs serve as monooxygenases during drug oxidation, receiving electrons from NADPH–P450 reductase. Reduced NADPH transfers a hydride ion to the FAD domain of the reductase, initiating electron transfer through the FAD and FMN to the adjacent CYP heme iron (Fe^3+^), generating ferrous iron (Fe^2+^). This electron transfer enables CYP to catalyze drug oxidation, generating the hydroxylated product and water as a byproduct. RH represents the substrate. ROH denotes the product of CYP-mediated oxidation. *FAD* flavin adenine dinucleotide, *FMN* flavin mononucleotide, *RH* substrate, *ROH* hydroxylated product

Cytochrome P450s (CYPs or P450s) are hemoproteins that contain a single heme prosthetic group within their active sites (Scott and Halpert 2005). The human CYP family plays a critical role in Phase I drug metabolism, predominantly by catalyzing monooxygenation reactions (Nebert and Dalton 2006). In these reactions, one oxygen atom is incorporated into the substrate, typically a hydrocarbon, while the other is reduced to form water. These reactions require the electron donor nicotinamide adenine dinucleotide phosphate (NADPH) and redox partner, such as NADPH–P450 reductase which transfers reducing equivalents to the CYP heme iron during catalysis (Durairaj and Li 2022) (Fig. 1). While CYPs share structural similarities and conserved mechanisms of action, their active sites vary considerably, accounting for substrate specificity (Table 1). CYPs are essential for metabolizing endogenous and exogenous substrates, including drugs, environmental chemicals, and natural products (Scott and Halpert 2005). Despite facilitating detoxification, CYP enzymes can also generate toxic metabolites, increasing cancer risk (Nebert and Dalton 2006). Specifically, reaction uncoupling in the CYP oxidation cycle produces reactive oxygen species (ROS) and dissociation of reaction intermediates, contributing to diseases, including cancer and cardiovascular diseases, through protein modifications, lipid peroxidation, and oxidative DNA damage (Moorthy and Veith 2018; Yamoune et al. 2024).

**Table 1:** Distribution and key characteristics of human CYP enzymes

CYP family		Tissue expression	Substrates*	Major role and characteristics	References
**CYP1**	1A1	Brain, gastrointestinal tract, heart, liver, lung, lymphocytes	PAHs, caffeine, eicosanoids, arachidonic acid	Induced by smoking. Catalyzes benzo[α]pyrene 3-hydroxylation	(Burkina et al. 2021; Kwon et al. 2021; Nakano et al. 2025)
	1A2	Liver	Aromatic amines, PAHs, caffeine, phenacetin, eicosanoids, arachidonic acid	Induced by smoking, meat, cruciferous vegetables, β-naphthoflavone. Catalyzes caffeine N^3^-demethylation, resorufin O-deethylation	(Miura et al. 2021; Kwon et al. 2021; Vilčková et al. 2023)
	1B1	Adrenal gland, brain (cortex), breast, bone marrow, heart, kidney, lung, ocular tissues, ovary, placenta, prostate, skin/keratinocytes, small intestine, testis	PAHs, dioxins, aflatoxin B1, estradiol, arachidonic acid, vitamin A, melatonin	Induced by AhR agonists (e.g., dioxin). Involved in xenobiotic and steroid metabolism, including 17β-estradiol (E_2_) 4-hydroxylation retinoic acid metabolism	(Zanger and Schwab 2013; Song et al. 2022a, b)
**CYP2**	2A6	Liver	Coumarin, 7-ethoxycoumarin, steroids, eicosanoids, arachidonic acid	Induced by barbiturates and dexamethasone. Catalyzes coumarin 7-hydroxylation	(Anzenbacher and Anzenbacherova 2001; Jiang et al. 2021)
	2B6	Liver, brain, kidney, intestine, endometrium, bronchoalveolar macrophages, peripheral blood lymphocytes, skin	Nicotine, arachidonic acid, lauric acid, steroids (17β-estradiol, estrone, ethinylestradiol, testosterone), environmental chemicals and pollutants	Induced by rifampicin, phenytoin, barbiturates, fasting/ energy restriction. Catalyzes 7-ethoxy-4-(trifluoromethyl)coumarin *O*-deethylation. 16α/16β-hydroxylation of steroids	(Chang et al. 2006; Wang and Tompkins 2008; Zanger and Klein 2013)
	2C8	Liver, kidney, intestine, brain, adrenal gland, arteries, duodenum, heart, lung, mammary gland, ovary, prostate, retina, testis, uterus	Unsaturated fatty acids, retinoic acid, arachidonic acid, environmental toxins, glucuronide metabolites, natural compounds (e.g., flavonoids, terpenes, alkaloids)	Induced by PXR ligands (e.g., phenobarbital, rifampicin). Catalyzes various oxidative reactions (e.g., hydroxylation, *N*-demethylation, *N*-deethylation)	(Schoch et al. 2004; Backman et al. 2016)
	2C9 (2C10)	Liver (predominant), intestine	Clinically important drugs (15–20%), including anticoagulants (warfarin), sulfonylurea antidiabetics (tolbutamide), NSAIDs	Induced by rifampicin, dexamethasone, carbamazepine, and aphenobartitone. Catalyzes tolbutamide 4′-hydroxylation	(Gómez-Tabales et al. 2020; Fekete et al. 2021)
	2C18	Liver (predominant), small intestine, brain, mammary gland	PAHs, retinoic acid, substrates of CYP2C8/2C9/2C19 (e.g., desomorphine, 4′-hydroxyphenytoin, naloxone)	Progesterone 16α-hydroxylation. 4′/5/6α/7-hydroxylation, *N*-dealkylation/deallylation/demethylation of drugs	(Yamazoe and Yoshinari 2024)
	2C19	Liver (predominant), duodenum	Arachidonic acid, prescribed drugs (8–10%), including pesticides, carcinogens	Detoxifies/inactivates carcinogens and bioactivates procarcinogens to reactive DNA-binding metabolites. Catalyzes 4- and 5-methyl hydroxylation	(Yadav et al. 2008; Sanford et al. 2013; Takayama et al. 2021; Vignaux et al. 2023)
	2D6	Liver, brain, intestinal tissue, lymphoid cells	Tyramine, 5-hydroxyindoleacetic acid, common drugs (~ 20%)	Catalyzes α-, 2-, 4-, 5-hydroxylation; *N*-dealkylation; *O*-demethylation	(Taylor et al. 2020; Dorne et al. 2002)
	2E1	Liver, brain, heart (mitochondria), lung, kidney, skin, skeletal muscle	Acetone, fatty acids, ethanol, nicotine, nitrosamines, aspartame, pollutants, drugs (e.g., acetaminophen, anesthetics, phenobarbital)	Activation of microsomal ethanol oxidizing system. linked to progressive metabolic diseases (e.g., obesity, diabetes)	(García-Suástegui et al. 2017; Ma et al. 2025)
	2F1	Respiratory tract (predominant)	Pulmonary toxicants (naphthalene, styrene, benzene, 3-methylindole)	Bioactivates pulmonary toxicants; induces tissue-specific toxicity	(Li et al. 2017)
	2J2	Heart (predominant), intestine, kidney, lung	n-6 and n-3 polyunsaturated fatty acids (arachidonic, linoleic, EPA, DHA), endocannabinoids, vitamin D analogs	Epoxidation of substrates	(Leow and Chan 2024)
	2R1	Liver, testis, kidney; broadly expressed in various tissues	Vitamin D	Major 25-hydroxylase of vitamin D. Suppressed by fasting, obesity, diabetes	(Elkhwanky et al. 2020)
	2U1	Thymus, brain	Eicosanoids, arachidonic acid	Hydroxylation of long-chain fatty acids	(Seliskar and Rozman 2007)
**CYP3**	3A4	Liver and intestine (≈30% of total P450s in liver; ≈80% in intestine)	> 50% of clinical drugs (e.g., antiestrogens, nitrogen mustards, taxanes, TKIs)	Phase I metabolism (e.g., oxidation, hydrolysis, reduction)	(Wang et al. 2023)
	3A5 3A7	Liver, gastrointestinal tract, intestine Fetal liver (main isoform), placenta	Drugs, carcinogens, steroid hormones (e.g., testosterone, progesterone), fatty acids retinoic acid, testosterone, dehydroepiandrosterone, drugs	Bimodal metabolism due to genetic polymorphism Shows 88% homology with CYP3A4 but generates different metabolite than CYP3A4 (e.g., testosterone metabolites of CYP3A4 and CYP3A7 are 6β-hydroxytestosterone and 2α-hydroxytestosterone, respectively.)	(Jiang et al. 2015; Matsumoto et al. 2021) (Topletz et al. 2019; Kabir et al. 2022)
**CYP4**	4A11	Kidney, liver	Medium/long-chain fatty acids (e.g., arachidonic, palmitate, lauric acid), endobiotics	Induced by clofibrate. Catalyzes ω- and (ω-1)-hydroxylation of fatty acids and 12′-hydroxylation of lauric acid. Linked to NAFLD	(Gao et al. 2020; Liu et al. 2021)
	4B1	Lung (predominant)	Fatty acids, hydrocarbons, xenobiotics (e.g., valproic acid)	Catalyzes ω-hydroxylation; oncogenic potential	(Liu et al. 2021)
	4F	Kidney, liver, small intestine, myeloid cells, seminal vesicles, epidermis, skin	Eicosanoids (arachidonic, leukotrienes, prostaglandin), vitamin K1, drugs (e.g., pafuramidine, fingolimod, ebastine, astemizole)	Catalyzes *O*-demethylation, oxidation, ω-, ω-1-, ω-2-hydroxylation	(Uehara et al. 2015; Uno et al. 2025)
**CYP5**		Platelet (primarily)	Prostaglandin H_2_	Functions as a thromboxane-A_2_ synthase and prostacyclin biosynthesis enzyme	(Das et al. 2014)
**CYP7**		Brain, liver (most abundant), ovary, prostate, colon, kidney, testis, small intestine	Cholesterol, steroid precursors (e.g., pregnenolone, dehydroepiandrosterone, oxysterols)	Hydroxylates cholesterol (rate-limiting step in bile acid synthesis); catalyzes 7α-hydroxylation of 25- and 27-hydroxycholesterol	(Yantsevich et al. 2014; Dzichenka et al. 2025)
**CYP8**		Vascular endothelial and smooth muscle cells (CYP8A1); liver (CYP8B1)	Steroids, cholesterol, lipids, drugs	Prostaglandin I_2_ synthase (CYP8A1, converts PGH_2_ → PGI_2_). sterol 12α-hydroxylase (CYP8B1) in bile acid synthesis	(Beltran-Sarmiento et al. 2016; Ding et al. 2023; Fleishman and Kumar 2024)
**CYP11**		Steroidogenic tissues, adrenal cortex, brain, gastrointestinal tract, immune system, skin (mitochondria)	Cholesterol, 11-deoxycortisol, 11-deoxycorticosterone	Catalyzes steroid hormone synthesis	(Omura 2006; Slominski et al. 2021)
**CYP17**		Adrenal cortex, testis, ovary	Pregnenolone, progesterone	Functions as steroid 17α-hydroxylase and 17/20-lyase	(Hakki and Bernhardt 2006)
**CYP19**		Ovary, placenta, testis, prostate, brain, bone, adipose tissue, gonad	Androstenedione, testosterone, 16-hydroxytestosterone	Aromatase (P450 aromatase); catalyzes estrogen synthesis (estrone, estradiol, estriol)	(Stocco 2012)
**CYP21**		Adrenal cortex	17-Hydroxyprogesterone	Steroid 21-hydroxylase. CYP21 deficiency → congenital adrenal hyperplasia	(Honour 2014)
**CYP24**		Kidney (mitochondria)		Catalyzes 24-hydroxylation of 1,25-dihydroxy vitamin D_3;_ regulates calcium homeostasis	(De Paolis et al. 2019; Fuchs et al. 2024)
**CYP26**		Adult liver, heart, pituitary gland, adrenal gland, placenta, brain		Catalyzes hydroxylation	(Seliskar and Rozman 2007; Manikandan and Nagini 2018; Nebert and Russell 2002)
**CYP27**		Liver, kidney, skin (mitochondria)		Catalyzes 27-hydroxylation of cholesterol, 1α- and 25-hydroxylation of vitamin D_3_, retinoid desaturation	(Child et al. 2020; Omura 2006)
**CYP39**		Liver	24-Hydroxycholesterol	Bile acid synthesis, 24-hydroxycholesterol 7α-hydroxylase	(Grabovec et al. 2019)
**CYP46**		Brain	Cholesterol	Catalyzes 24-hydroxylation	(Cataldi et al. 2023)
**CYP51**		Ovary, adrenal gland, prostate, liver, kidney, lung, testis	Lanosterol	Functions as lanosterol 14α-demethylase in cholesterol synthesis	(Seliskar and Rozman 2007; Nebert and Russell 2002)

^*^Substrate examples are selectively included. Specific drug names are provided only in exceptional cases

NSAIDs, non-steroidal anti-inflammatory drugs; PXR, pregnane X receptor; PUFAs, polyunsaturated fatty acids; EPA, eicosapentaenoic acid; DHA, docosahexaenoic acid; DHEA, dehydroepiandrosterone; NAFLD, non-alcoholic fatty liver disease

Translational cancer research has shifted from conventional cytotoxic chemotherapy toward targeted prodrug approaches to improve anticancer drug efficacy (Yang et al. 2020; Lee et al. 2024). This strategy exploits unique patterns of CYP expression and activity to enhance the precision and effectiveness of cancer treatments (Singh et al. 2023).

Additionally, CYPs are implicated in the pathogenesis of non-neoplastic diseases. In the liver, CYP-mediated drug metabolism generates toxic metabolites that cause hepatotoxicity (Wu et al. 2017; Shin et al. 2023). In cardiovascular tissues, including the heart, endothelium, and vascular smooth muscle, CYPs contribute to cardiovascular homeostasis by metabolizing endogenous compounds, such as epoxyeicosatrienoic acids (EETs), hydroxyeicosatetraenoic acids (HETEs), prostaglandins, aldosterone, and sex hormones. Their activity correlates with various cardiovascular diseases, including hypertension, stroke, and arrhythmia (Elbekai and El-Kadi 2006). Hence, targeting CYPs has emerged as a novel drug discovery approach, with ongoing investigations into CYP inhibition and induction by established drugs.

Drugs interact with the CYP system via inhibition or induction (Hakkola et al. 2020). Inhibitors reduce CYP metabolic activity, depending on dosage and enzyme-binding capacity (Lynch and Price 2007). Certain drugs are metabolized or inhibited by the same enzyme, while others are metabolized by one enzyme and inhibited by another (Kotlyar et al. 2005). Clinically, drug combinations may exploit CYP inhibition for therapeutic benefit (Ramanarayanan and Scarpace 2007; Guo et al. 2018). Modulating CYP activity leads to clinically relevant drug–drug interactions (DDIs), causing unexpected adverse reactions or altered therapeutic effects (Scott and Halpert 2005; Lynch and Price 2007; Hakkola et al. 2020).

Reversible CYP inhibitors disrupt the catalytic cycle before forming activated oxygen intermediates. Conversely, inhibitors acting during or after the formation of activated oxygen intermediates are classified as quasi-irreversible or irreversible (Kamel and Lamsabhi 2020). Reversible CYP inhibition is considered the primary mechanism underlying DDIs (Hollenberg 2002). Assessing the consequences of the interactions between CYP inhibitors and drugs metabolized by targeted CYPs has substantial clinical relevance (Kilford et al. 2022).

Therefore, this review aims to examine the roles of CYP isoforms in various diseases, including cancer, and their significance in drug development. Further, Food and Drug Administration (FDA)-approved CYP inhibitors and recent advancements in novel inhibitor research are discussed. The findings could provide a comprehensive understanding of CYP-targeted therapies and their clinical applications.

## Role of cytochrome P450-targeted inhibitors in cancer

**Fig. 2:**
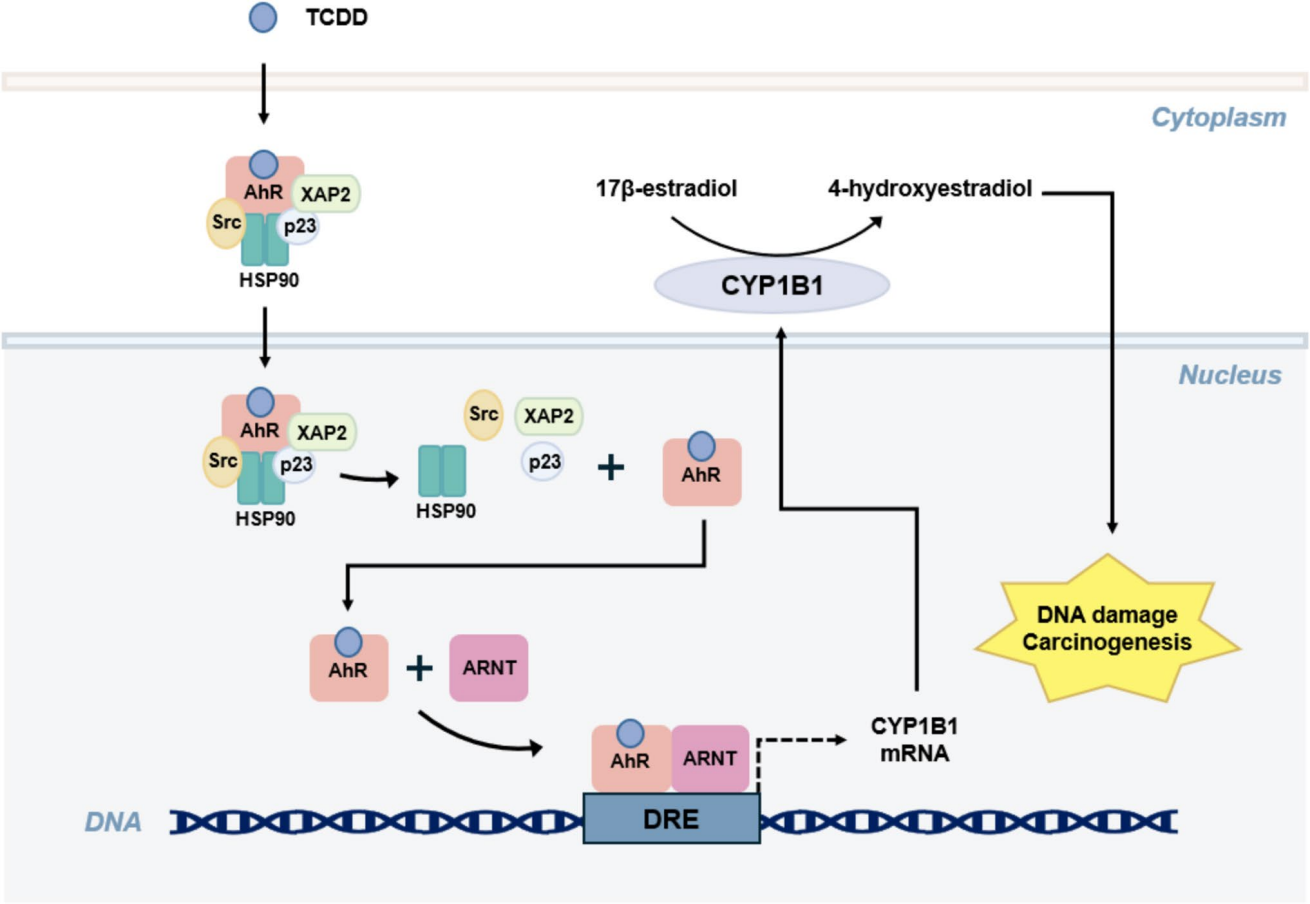
CYP1B1-mediated carcinogenesis via AhR activation by dioxin. In the cytoplasm, inactive AhR is bound to a chaperone complex that includes a HSP90 dimer and XAP2. Upon ligand binding, such as TCDD, AhR translocates into the nucleus where it dissociates from the chaperone complex and forms a heterodimer with ARNT. This heterodimer binds to the DRE, initiating CYP1B1 transcription. CYP1B1, localized at the endoplasmic reticulum membrane, catalyzes the hydroxylation of 17β-estradiol to form 4-hydroxyestradiol. This metabolite facilitates carcinogenesis by inducing DNA adduct formation in the nucleus. *ARNT* AhR nuclear translocator, *DRE* dioxin-responsive element, *ER* endoplasmic reticulum, *HSP90* heat shock protein 90, *TCDD* 2,3,7,8-tetrachlorodibenzo-p-dioxin, *XAP2* X-associated protein 2

CYPs are crucial for the pathogenesis of renal, lung, breast, ovarian, prostate, and hematological malignancies. Specifically, cytochrome P450 1B1 (CYP1B1), which hydroxylates estrogen, contributes to cancer initiation through procarcinogen metabolism (Murray et al. 2014; Kwon et al. 2021) (Fig. 2). Consequently, extensive research has been conducted to investigate the potential of existing anticancer agents to inhibit CYP activity, identifying candidates targeting these enzymes (Table 2).

**Table 2:** Types of anticancer agents and their target cytochrome P450 enzymes based on cancer types

Cancer type	Inhibitor	Cytochrome P450 isoforms	References
Breast	Anastrozole	CYP1A2, CYP2C9, CYP3A4	(Eissa et al. 2023)
	Capecitabine	CYP2C9	(Guengerich 2022)
	Docetaxel	CYP1B1	(Rodriguez-Antona and Ingelman-Sundberg 2006; Rochat et al. 2001)
	Doxorubicin	CYP1B1, CYP2C8, CYP2D6	(Guengerich 2022; Lai et al. 2009; Rochat et al. 2001)
	Exemestane	CYP2C8	(Lai et al. 2009)
	Letrozole	CYP2A6, CYP2B6, CYP2C19	(Rodriguez-Antona and Ingelman-Sundberg 2006; Jeong et al. 2009; Buzdar et al. 2002)
	Mitoxantrone	CYP1B1	(Rodriguez-Antona and Ingelman-Sundberg 2006; Rochat et al. 2001)
	Ribociclib	CYP3A, CYP1A2	(Guengerich 2022; Bellet et al. 2019)
	Paclitaxel	CYP1B1, CYP2C8	(Rodriguez-Antona and Ingelman-Sundberg 2006; Rochat et al. 2001)
	Palbociclib	CYP3A	(Bellet et al. 2019)
	Tamoxifen	CYP3A, CYP1B1, CYP2C8, CYP2C9	(Rodriguez-Antona and Ingelman-Sundberg 2006; Lai et al. 2009; Rochat et al. 2001; Zhao et al. 2002; Boruban et al. 2006)
	Thiotepa	CYP2B6	(Rodriguez-Antona and Ingelman-Sundberg; 2006; Rae et al. 2002)
	Toremifene	CYP2C9	(Turpeinen et al. 2013)
Prostate	Flutamide	CYP1B1	(Rochat et al. 2001)
Ovary	Rucaparib	CYP1A2, CYP2C9, CYP2D6, CYP2C19	(Guengerich 2022)
Non-small cell lung	Erlotinib	CYP3A	(Dong et al. 2011)
	Gefitinib	CYP3A, CYP2D6	(Rodriguez-Antona and Ingelman-Sundberg 2006; Wang et al. 2021; Semba et al. 2020)
	Vinorelbine	CYP3A	(Rodriguez-Antona and Ingelman-Sundberg 2006; Kajita et al. 2000)
Colon	Regorafenib	CYP3A4	(Guengerich 2022)
Hematologic	Cytarabine	CYP3A	(Jain 2005)
	Daunomycin	CYP1B1	(Rochat et al. 2001)
	Enasidenib	CYP2C9, CYP2D6, CYP2C8, CYP2C19	(Cheng et al. 2022)
	Idarubicin	CYP2D6	(Rodriguez-Antona and Ingelman-Sundberg 2006; Jain 2005)
	Idelalisib	CYP3A	(Guengerich 2022)
	Imatinib	CYP3A	(Rodriguez-Antona and Ingelman-Sundberg 2006; Wang et al. 2021)
	Interferon	CYP1A2	(Guengerich 2022)
	Panobinostat	CYP2D6	(Guengerich 2022)
	Teniposide	CYP2C9	(Guengerich 2022)

### Hormone-induced cancers

#### Cytochrome P450 family 1 (CYP1) inhibitors

CYP1 family members, including cytochrome P450 1A1 (CYP1A1), cytochrome P450 1A2 (CYP1A2), and CYP1B1, are crucial for sex-hormone metabolism and are present in extrahepatic tissues, including breast, ovary, prostate, uterus, lung, muscle, and placenta (Go et al. 2015; Kwon et al. 2021). These enzymes participate in estrogen (E2, 17β-estradiol) metabolism, producing different major metabolites, which include: CYP1A1 and CYP1A2, which predominantly produce 2-hydroxyestradiol, while CYP1B1 produces 4-hydroxyestradiol. These catecholestrogen metabolites are key contributors to carcinogenesis (Kwon et al. 2021). CYP1 enzymes are regulated by the estrogen and aryl hydrocarbon receptors (AhR) (Go et al. 2015) and metabolize dioxins and polycyclic aromatic hydrocarbons (PAHs), including 2,3,7,8-tetrachlorodibenzo-p-dioxin (TCDD) (Shimada and Fujii-Kuriyama 2004). Additionally, these enzymes bioactivate carcinogens, particularly heterocyclic amines, aromatic amines, and nitropolycyclic hydrocarbons (Chun and Kim 2003). Consequently, CYP1 metabolizes hormone-induced cancer treatments and serves as a target for anticancer drugs (Go et al. 2015; Fabris et al. 2023).

The ethoxyresorufin O-deethylase (EROD) assay is commonly used to measure inhibition of CYP1B1 activity by anticancer drugs (Rochat et al. 2001). Competitive inhibitors include flutamide, mitoxantrone, docetaxel, and paclitaxel, with *K*_*i*_ values of 1.0 μM, 11.6 μM, 28.0 μM, and 31.6 μM, respectively. Noncompetitive or mixed inhibitors comprise daunomycin, doxorubicin, and tamoxifen, with *K*_*i*_ values of 2.1 μM, 2.6 μM, and 5.0 μM, respectively (Rochat et al. 2001).

Flutamide, an androgen-receptor antagonist used in prostate cancer therapy, inhibits CYP1A1, CYP1A2, and CYP1B1. CYP1B1 catalyzes 2-hydroxylation of flutamide, contributing to drug resistance, whereas flutamide competitively inhibits CYP1A1 (*K*_*i*_ = 10.3 ± 1.4 μM), CYP1A2 (*K*_*i*_ = 1.4 ± 0.3 μM), and CYP1B1 (*K*_*i*_ = 1.0 ± 0.1 μM) (Rochat et al. 2001; Singh et al. 2023).

#### Aromatase (CYP19) inhibitors; anastrozole, letrozole, exemestane

Estrogen plays a critical role in breast cancer development (Smith and Dowsett 2003). Aromatase, encoded by the *CYP19* gene (Mallikarachchi et al. 2024), synthesizes estrone from androstenedione and 17β-estradiol from testosterone (Miller 2003). Aromatase inhibitors can significantly suppress plasma estrogen levels in postmenopausal women (Miller 2003; Smith and Dowsett 2003). Nonsteroidal aromatase inhibitors, including anastrozole, vorozole, letrozole, and fadrozole, were developed to address limitations of conventional steroidal aromatase inhibitors such as exemestane, improving oral bioavailability and favorable tolerability (Bhatia and Thareja 2024; Karaer et al. 2004). Anastrozole and letrozole are competitive, reversible third-generation inhibitors approved to treat progressive, postmenopausal, estrogen-responsive breast cancer; both exhibit high efficacy and selectivity for CYP19 (Geisler 2011). In vitro*,* anastrozole inhibits hepatic CYP1A2, cytochrome P450 2C9 (CYP2C9), and cytochrome P450 3A (CYP3A) activities, but this effect is much weaker than that on aromatase and does not occur at therapeutic concentrations (Linardi et al. 2017; Eissa et al. 2023).

CYP2A6, cytochrome P450 3A4 (CYP3A4), and cytochrome P450 3A5 (CYP3A5) metabolize letrozole to produce the human metabolite 4,4'-methanol-bisbenzonitrile (Jeong et al. 2009; Keating 2009). Letrozole strongly inhibits CYP2A6 and weakly inhibits cytochrome P450 2C19 (CYP2C19) (Burk and Wojnowski 2004; Jeong et al. 2009), while its metabolite inhibits cytochrome P450 2B6 (CYP2B6) and CYP2C19 (Jeong et al. 2009). Type II aromatase inhibitors (anastrozole and letrozole) reversibly bind and inhibit aromatase. Conversely, exemestane—a type I steroidal aromatase inhibitor—irreversibly inactivates aromatase as a structural analog of androstenedione (Sobral et al. 2016; Kim et al. 2024) and also functions as a reversible inhibitor of cytochrome P450 2C8 (CYP2C8) (Lai et al. 2009). In vitro, exemestane inhibits CYP2C8-catalyzed N-deethylation of amodiaquine, with an IC_50_ value of 13.5 μM (Lai et al. 2009).

#### Cyclin-dependent kinase 4 and 6 (CDK4/6) inhibitors: ribociclib, palbociclib

Endocrine therapy has traditionally been used to treat patients with hormone receptor (HR)-positive breast cancer. However, resistance to endocrine treatment has been observed in those treated with CDK4/6 inhibitors (Pandey et al. 2019). These inhibitors block CDK4/6 (Pernas et al. 2018) and inhibit retinoblastoma protein (Rb) phosphorylation, preventing cell cycle progression from the G1 to S phase. This ultimately results in cell cycle arrest and reduced proliferation (Pernas et al. 2018; Pandey et al. 2019). Three CDK4/6 inhibitors— palbociclib (PD0332991), ribociclib (LEE011), and abemaciclib (LY835219)—are Food and Drug Administration (FDA)-approved for ER-positive metastatic breast cancer and as first-line therapy in combination with nonsteroidal aromatase inhibitors for postmenopausal women (Murphy 2019).

CYP3A isozymes, such as CYP3A4, CYP3A5, cytochrome P450 3A7 (CYP3A7), and cytochrome P450 3A43 (CYP3A43), are clinically important owing to their broad substrate specificity and interindividual variability in expression levels (Burk and Wojnowski 2004; Klyushova et al. 2022). These enzymes metabolize both exogenous chemical drugs and endogenous compounds such as steroids. The CYP3A subfamily is also a major contributor to DDIs (Burk and Wojnowski 2004). Particularly, CYP3A4—expressed primarily in the liver and intestine—metabolizes approximately 50% of all clinically used drugs and several procarcinogens (Ashida et al. 2017). Accordingly, CYP3A4 is often involved in DDIs because it can be induced or inhibited by various anticancer drugs (Tian and Hu 2014). However, co-administration of CYP3A4 inhibitors with drugs metabolized by this isozyme often elevates plasma concentrations, thereby increasing the risk of drug-related toxicity (Tian and Hu 2014).

Among the CDK4/6 inhibitors, palbociclib and ribociclib function as both substrates and inhibitors of CYP3A4 (Bellet et al. 2019; Guengerich 2022). Palbociclib is a weak inhibitor of CYP3A4, while ribociclib acts as a moderate inhibitor at 400 mg/day and a strong inhibitor at 600 mg/day (Bellet et al. 2019). Owing to its inherent inhibitory effect on CYP3A4, ribociclib can elevate serum levels of CYP3A4 substrates (Bellet et al. 2019). Consequently, both palbociclib and ribociclib reduce CYP3A4metabolic capacity, leading to drug accumulate in the systemic circulation and potential toxicity (Bellet et al. 2019). Caution is therefore required when these inhibitors are co-administered with other CYP3A4-metabolized drugs.

#### Others: abiraterone, orteronel

Cytochrome P450 17A1 (CYP17A1), also known as 17α-monooxygenase or 17α-hydroxylase/17,20-lyase/17,20-desmolase, is essential in steroidogenesis, including androgen biosynthesis, and contributes significantly to prostate cancer pathogenesis (Aherrahrou et al. 2020; Cao et al. 2020). Abiraterone, administered as prodrug abiraterone acetate, is the sole FDA-approved CYP17A1 inhibitor and demonstrates synergistic efficacy when combined with androgen deprivation therapy (ADT) in patients with prostate cancer (Cheong et al. 2020; Wróbel et al. 2023). Despite the high affinity of abiraterone for CYP17A1, it has been associated with various adverse effects arising from off-target interactions with other CYPs such as cytochrome P450 21A2 (CYP21A2) (Udhane et al. 2016). To address these limitations, orteronel (TAK-700), a novel nonsteroidal, reversible inhibitor, has been developed with greater specificity for cytochrome P450 17,20-lyase (CYP17,20-lyase) than 17α-hydroxylase (IC_50_ = 19 nM). Orteronel selectively inhibits androgen synthesis in prostate cancer cells without inducing secondary mineralocorticoid excess syndrome as a side effect (Cao et al. 2020; Agarwal et al. 2022).

### Lung cancer

The lungs are a primary site of exposure to inhaled toxicants from tobacco smoke, including mutagenic and carcinogenic compounds, which are metabolized by CYPs into carcinogenic intermediates (Chen et al. 2024a, b; Bellanca et al. 2025). The CYP3A subfamily, including CYP3A4, metabolizes PAHs and procarcinogens from tobacco. Specifically, CYP3A4 and CYP3A5 activate benzo[*a*]pyrene, and N9-nitrosonornicotine, and contribute to lung cancer through induction of genetic alteration (Islam MS et al. 2014).

#### Erlotinib

Erlotinib, a first-generation epidermal growth factor receptor (EGFR) tyrosine kinase inhibitor (TKI), is approved for the treatment of advanced or metastatic non-small-cell lung cancer (NSCLC) in the United States and Switzerland (Ling et al. 2006). It is primarily metabolized by CYP3A4 and CYP1A2, with additional contribution from extrahepatic CYP1A1 (Ling et al. 2006). CYP3A4 and CYP3A5 convert erlotinib into its active metabolite OSI-420, which inhibits EGFR tyrosine kinase activity (Fukudo et al. 2013).

Several DDI studies have characterized CYP3A4, a key drug-metabolizing enzyme. Research on erlotinib interactions with CYP3A4 inhibitors, including ketoconazole, clarithromycin, and voriconazole, suggests that these inhibitors can cause erlotinib toxicity by inhibiting CYP3A4 (Ramanarayanan and Scarpace 2007). Conversely, erlotinib also inhibits CYP3A4 by more than 50%, consistent with competitive inhibition (*K*_*i*_ = 14.1 ± 4.3 μM) (Dong et al. 2011). This inhibition is substrate-dependent, while time-dependent inhibition affects DDIs independently of substrate (Dong et al. 2011).

#### Gefitinib

TKIs are effective long-term oral treatments for cancer; however, their concurrent administration with other drugs increases the risk of DDIs (Shao et al. 2014). Clinical DDIs often occur owing to the inhibition or induction of CYP enzymes (Li et al. 2009). In vitro studies show that TKIs such as imatinib, sunitinib, and gefitinib may irreversibly inhibit CYP3A enzymes, potentially contributing to DDIs (Wang et al. 2021).

Gefitinib, a second-generation TKI, is used to treat advanced NSCLC with EGFR mutations (Dhillon 2015). CYP3A inhibition was evaluated using testosterone as the substrate, with 6β-OH-testosterone quantified as the metabolite. A minor initial inhibition increased after a 30-min incubation with NADPH, suggesting that gefitinib inhibits CYP3A in an NADPH-dependent manner (Wang et al. 2021).

Furthermore, Gefitinib inhibits CYP2D6 variants in vitro (Fang et al. 2017; Semba et al. 2020). CYP2D6 is a highly polymorphic metabolic enzyme, with over 100 genetic variants, including CYP2D6.2 (Arg296Cys, Ser486Thr), CYP2D6.10 (Pro34Ser, Ser486Thr), and CYP2D6.39 (Ser486Thr), which are associated with significant alterations in enzyme activity (Sakuyama et al. 2008). The inhibitory effect of gefitinib on CYP2D6 variants was quantified by evaluating the O-demethylation of dextromethorphan (Semba et al. 2020). Intrinsic clearance (*V*_*max*_/*K*_*M*_) values for CYP2D6.2 (Arg296Cys and Ser486Thr), CYP2D6.10 (Pro34Ser and Ser486Thr), and CYP2D6.39 (Ser486Thr) were 0.57-, 0.038-, and 0.47-folds higher than that of CYP2D6.1 (wild type), respectively (Semba et al. 2020). Using a mixed inhibition model, the inhibitory efficacy of gefitinib varied across CYP2D6 variants (Fang et al. 2017; Semba et al. 2020). The *K*_*i*_ values for CYP2D6.2, CYP2D6.10, and CYP2D6.39 were 40%, 150%, and 50% higher than that of the wild-type enzyme, respectively, indicating reduced susceptibility to inhibition by gefitinib (Semba et al. 2020). These findings suggest that CYP2D6 genetic diversity may contribute to variability in the potency of CYP2D6-mediated DDIs with gefitinib among individuals (Semba et al. 2020).

### Hematologic cancer

CYPs are associated with the development of leukemia and lymphoma (Nagai et al. 2002; Sandoval et al. 2023). CYP1A1, CYP1B1, CYP2A6, CYP2A7, CYP2D6, and CYP2E1 are expressed in myeloid leukemia (U937, HL-60, and K562) and lymphoid (BALL-1, MOLT-4, and Jurkat) cell lines (Nagai et al. 2002). Additionally, the CYP2E1*5 allele, a variant associated with CYP2E1 gene polymorphisms, increases the risk of acute myeloid and lymphoblastic leukemia (Sandoval et al. 2023).

#### Enasidenib

Enasidenib, an oral inhibitor of mutant isocitrate dehydrogenase-2 (IDH2) protein, was approved in the United States in 2017 to treat adult patients with relapsed or refractory (R/R) acute myeloid leukemia (AML) with an IDH2 mutation (Pollyea et al. 2019; Cheng et al. 2022). Nonclinical studies demonstrate that enasidenib directly inhibits CYP2C8, CYP2C9, CYP2C19, and CYP2D6 at concentrations comparable to those observed in patients with AML at steady state (Cheng et al. 2022).

#### Imatinib

Imatinib is a first-generation small-molecule kinase inhibitor that targets the BCR-ABL protein tyrosine kinase and is primarily used to treat chronic myeloid leukemia (CML) by inhibiting its aberrant kinase activity (Peng et al. 2005; Wang et al. 2021). The effects of imatinib on DDIs and CYP3A enzyme activities are well-characterized (Peng et al. 2005; Haouala et al. 2011; Wang et al. 2021). Primarily metabolized by CYP3A4 and CYP3A5, imatinib competitively inhibits the metabolism of drugs that are substrates for CYP2C9, CYP2C19, CYP2D6, and CYP3A4/5, leading to increased plasma drug concentrations (Peng et al. 2005; Rodriguez-Antona and Ingelman-Sundberg 2006; Haouala et al. 2011). The interaction between imatinib and immunosuppressive cyclosporine, both metabolized by CYP3A and inhibitors of CYP3A4, may alter hepatic drug metabolism through competitive inhibition of CYP3A4 (Peng et al. 2005). Imatinib can inhibit drug transporters and CYP3A4, thereby increasing the intestinal absorption of cyclosporine—a substrate of CYP3A4 and the drug transporter P-glycoprotein—leading to enhanced pharmacological effects and potential toxicity (Haouala et al. 2011). Molecular docking analyses comparing imatinib, gefitinib, and sunitinib indicated that imatinib binds most strongly to the active site of CYP3A4 in its flattest conformation with the optimal binding mode. This property makes imatinib the most potent CYP3A4 inhibitor among TKIs, conferring the highest risk of DDIs (Wang et al. 2021).

## Role of drugs targeting cytochrome P450 in non-neoplastic human diseases

CYPs are associated with central nervous system disorders, including stroke, Alzheimer’s disease, Parkinson's disease, epilepsy, multiple sclerosis, and psychiatric conditions such as anxiety and depression (Liu et al. 2004a, b). Furthermore, hepatic CYPs regulate diabetes, obesity, infection, and inflammatory diseases (Cheng and Morgan 2001). Numerous studies have investigated the potential of targeting and inhibiting CYPs using FDA-approved drugs (Table 3). Therefore, understanding the role of these drugs in non-cancerous diseases is essential.

**Table 3:** Drugs used for common non-cancer diseases and symptoms, with their target cytochrome P450 enzymes

Disease class	Inhibitor	Cytochrome P450 isoforms	References
ADHD	Atomoxetine	CYP3A4	(Guengerich 2022)
Allergy	Cetirizine	CYP2B6	(Walsky et al. 2006)
	Chlorpheniramine	CYP2D6, CYP2B6	(Guengerich 2022; He et al. 2002; Walsky et al. 2006)
	Clemastine	CYP2D6	(Guengerich 2022)
	Cyclizine	CYP2D6, CYP2C9	(He et al. 2002)
	Desloratadine	CYP2B6	(Walsky et al. 2006)
	Diphenhydramine	CYP2D6	(Guengerich 2022; He et al. 2002)
	Fexofenadine	CYP2B6	(Walsky et al. 2006)
	Hydroxyzine	CYP2D6, CYP2B6	(Guengerich 2022; Walsky et al. 2006)
	Loratadine	CYP2C8, CYP2B6	(Lai et al. 2009; Walsky et al. 2006)
	Olopatadine	CYP2B6	(Walsky et al. 2006)
	Promethazine	CYP2D6, CYP2C9	(Guengerich 2022; He et al. 2002)
	Terfenadine	CYP2C8, CYP2B6	(Lai et al. 2009; Walsky et al. 2006)
	Tripelennamine	CYP2D6	(Guengerich 2022; He et al. 2002)
Arrhythmia	Amiodarone	CYP1A2, CYP2C8, CYP2C9, CYP2D6, CYP3A4	(Guengerich 2022; Lai et al. 2009)
	Quinidine	CYP2D6	(Guengerich 2022)
Asthma	Furafylline	CYP1A2	(Guengerich 2022)
	Montelukast	CYP2C8, CYP2C9	(Lai et al. 2009)
	Pranlukast	CYP2C9	(Liu et al. 2004a, b)
	Salmeterol	CYP2C8	(Lai et al. 2009)
	Zafirlukast	CYP1A2, CYP2C8, CYP2C9, CYP3A4, CYP2C19, CYP2D6	(Guengerich 2022; Lai et al. 2009; Liu et al. 2004a, b)
	Zileuton	CYP2C8	(Lai et al. 2009)
Atopic eczema	Crisaborole	CYP1A2, CYP2C9	(Guengerich 2022)
Bacterial infection	Cefuroxime axetil	CYP2C8	(Lai et al. 2009)
	Ciprofloxacin	CYP3A4, CYP1A2	(Guengerich 2022; Granfors et al. 2004)
	Clarithromycin	CYP3A4	(Guengerich 2022; Tian and Hu 2014)
	Erythromycin	CYP3A4	(Guengerich 2022; Tian and Hu 2014; Zhou 2008)
	Isoniazid	CYP3A4, CYP1A2, CYP2A6, CYP2C19, CYP2C8	(Wen et al. 2002; Desta et al. 2001; Lai et al. 2009)
	Metronidazole	CYP2C9	(Guengerich 2022)
	Norfloxacin	CYP3A4	(Guengerich 2022)
	Quinolones	CYP1A2	(Guengerich 2022; Zhou et al. 2010)
	Sulfamethoxazole	CYP2C9	(Guengerich 2022)
	Sulfaphenazole	CYP2C9, CYP2C8	(Guengerich 2022; Lai et al. 2009)
	Telithromycin	CYP3A4	(Guengerich 2022)
	Trimethoprim	CYP2C8	(Lai et al. 2009)
Depression	Bupropion	CYP2D6	(Kotlyar et al. 2005)
	Citalopram	CYP2D6, CYP1A2, CYP2C19	(Guengerich 2022)
	Clomipramine	CYP2D6	(Guengerich 2022)
	Desipramine	CYP2C8	(Lai et al. 2009)
	Doxepin	CYP2D6	(Guengerich 2022)
	Duloxetine	CYP2D6	(Guengerich 2022)
	Escitalopram	CYP2D6	(Guengerich 2022)
	Fluoxetine	CYP2D6, CYP2C19, CYP2C8	(Guengerich 2022; Lai et al. 2009)
	Fluvoxamine	CYP3A4, CYP1A2, CYP2C9, CYP2C19	(Guengerich 2022)
	Moclobemide	CYP2C19, CYP2D6, CYP1A2	(Borowicz-Reutt et al. 2021)
	Nefazodone	CYP3A4, CYP2C8	(Guengerich 2022; Lai et al. 2009)
	Norfluoxetine	CYP3A4	(Guengerich 2022)
	Nortriptyline	CYP2C8	(Lai et al. 2009)
	Paroxetine	CYP2D6, CYP2C9	(Guengerich 2022)
	Phenelzine	CYP2C8	(Lai et al. 2009)
	Sertraline	CYP2D6, CYP2C9, CYP2C8	(Guengerich 2022; Lai et al. 2009)
	Tranylcypromine	CYP2C8, CYP2C19, CYP2D6, CYP2C9, CYP2A6	(Lai et al. 2009; Salsali et al. 2004; Tanner and Tyndale 2017)
Diabetes	Glyburide	CYP2C8	(Lai et al. 2009)
	Pioglitazone	CYP2C8	(Lai et al. 2009)
	Rosiglitazone	CYP2C8	(Lai et al. 2009)
	Troglitazone	CYP2C8	(Lai et al. 2009)
Drug intoxication	Disulfiram	CYP2E1	(Xiong et al. 2025)
Epilepsy	Carbamazepine	CYP2C19	(Lakehal et al. 2002)
	Felbamate	CYP2C19	(Guengerich 2022)
	Oxcarbazepine	CYP2C19	(Guengerich 2022; Soskin et al. 2010)
	Topiramate	CYP2C19	(Soskin et al. 2010; Bialer et al. 2004)
Gout	Benzbromarone	CYP2C9, CYP3A4	(Locuson et al. 2003; Tang et al. 2021)
	Lesinurad	CYP2C8, CYP2C9	(Shen et al. 2019)
	Probenicid	CYP2C9, CYP2C19	(Guengerich 2022)
HCV	Boceprevir	CYP3A4	(Guengerich 2022)
	Telaprevir	CYP3A4	(Guengerich 2022)
Hypercholesterolemia	Fluvastatin	CYP2C8, CYP2C9	(Guengerich 2022; Lai et al. 2009)
Hyperlipidemia	Atorvastatin	CYP2C8	(Lai et al. 2009)
	Fenofibrate	CYP2C8, CYP2C9	(Guengerich 2022; Lai et al. 2009)
	Gemfibrozil	CYP2C8, CYP2C9	(Lai et al. 2009; Tornio et al. 2017; Wen et al. 2001)
	Lovastatin	CYP2C8, CYP2C9	(Guengerich 2022; Lai et al. 2009)
	Simvastatin	CYP2C8	(Lai et al. 2009)
Hyperparathyroidism	Cinacalcet	CYP2D6	(Guengerich 2022)
Hypertension	Amlodipine	CYP2C8	(Lai et al. 2009)
	Candesartan	CYP2C8	(Lai et al. 2009)
	Carvedilol	CYP2C8	(Lai et al. 2009)
	Diltiazem	CYP2C8, CYP3A4	(Zisaki et al. 2015; Guengerich 2022; Lai et al. 2009)
	Felodipine	CYP2C8	(Lai et al. 2009)
	Irbesartan	CYP2C8	(Lai et al. 2009)
	Losartan	CYP2C8	(Lai et al. 2009)
	Mibefradil	CYP1A2, CYP2C8	(Guengerich 2022)
	Nicardipine	CYP2C8	(Lai et al. 2009)
	Nifedipine	CYP2C8	(Lai et al. 2009)
	Verapamil	CYP3A4, CYP2C8	(Guengerich 2022)
Hypotension	Midodrine	CYP2D6	(Guengerich 2022)
Hypothyroidism	Levothyroxine	CYP2C8	(Lai et al. 2009)
Inflammation	Celecoxib	CYP2D6, CYP2C8	(Guengerich 2022; Lai et al. 2009)
	Dexamethasone	CYP2C8	(Lai et al. 2009)
	Indomethacin	CYP2C19	(Guengerich 2022)
	Methylprednisolone	CYP2C8	(Lai et al. 2009)
	Mometasone furoate	CYP2C8	(Lai et al. 2009)
	Phenylbutazone	CYP2C9	(Guengerich 2022)
	Rofecoxib	CYP1A2	(Karjalainen et al. 2006)
	Triamcinolone	CYP2C8	(Lai et al. 2009)
	Valdecoxib	CYP2C8	(Lai et al. 2009)
Malaria	Artemisinin	CYP1A2, CYP2C19, CYP2D6, CYP3A4	(Chamboko et al. 2023)
	Chloroquine	CYP2D6	(Chamboko et al. 2023)
	Halofantrine	CYP2D6	(Guengerich 2022)
	Hydroxychloroquine	CYP3A4, CYP2D6	(Paludetto et al. 2023)
	Quinine	CYP2C8	(Lai et al. 2009)
Mycosis	Caspofungin	CYP3A4	(Jain 2005)
	Chloramphenicol	CYP3A4, CYP2C19	(Guengerich 2022)
	Clotrimazole	CYP2C8	(Lai et al. 2009)
	Fluconazole	CYP3A4, CYP2C9, CYP2C19	(Guengerich 2022; Akamatsu et al. 2023)
	Itraconazole	CYP3A4	(Guengerich 2022; Tian and Hu 2014)
	Ketoconazole	CYP3A4, CYP2C19, CYP2C8	(Lai et al. 2009; Tian and Hu 2014; Boulenc et al. 2016; Greenblatt et al. 2011)
	Nystatin	CYP2C8	(Lai et al. 2009)
	Terbinafine	CYP2D6	(Nahid et al. 2025)
	Thiabendazole	CYP1A2	(Bapiro et al. 2005)
	Voriconazole	CYP3A4, CYP2B6, CYP2C9, CYP2C19	(Guengerich 2022; Shibata et al. 2021)
Nausea & vomiting	Aprepitant	CYP3A4	(Guengerich 2022)
	Levomepromazine	CYP2D6	(Guengerich 2022)
	Metoclopramide	CYP2D6	(Guengerich 2022)
	Netupitant	CYP3A4	(Guengerich 2022)
	Palonosetron	CYP2D6	(Guengerich 2022)
	Rolapitant	CYP2D6	(Guengerich 2022)
Osteoporosis	Raloxifene	CYP2C8	(Lai et al. 2009)
Overactive bladder	Oxybutynin	CYP2C8	(Lai et al. 2009)
Pain relief	Methadone	CYP2D6	(Guengerich 2022)
Peptic ulcers	Cimetidine	CYP1A2, CYP2C9, CYP2D6, CYP2C19, CYP3A4/5	(Guengerich 2022; Wendl et al. 2022)
	Esomeprazole	CYP2C19, CYP3A4/5	(Guengerich 2022)
	Lansoprazole	CYP2C19	(Guengerich 2022)
	Omeprazole	CYP2C19, CYP3A4/5	(Guengerich 2022)
	Pantoprazole	CYP2C19, CYP3A4/5	(Guengerich 2022)
	Rabeprazole	CYP2C8	(Lai et al. 2009)
Psoriasis	Methoxsalen	CYP1A2, CYP2A6, CYP2A13	(Palacharla et al. 2019; Sharma et al. 2024)
Schizophrenia	Chlorpromazine	CYP2D6	(Guengerich 2022)
	Clozapine	CYP2D6	(Edinoff et al. 2021)
	Fluphenazine	CYP1A2, CYP2D6	(Daniel et al. 2001)
	Haloperidol	CYP2D6	(Guengerich 2022)
	Perphenazine	CYP1A2, CYP2D6	(Guengerich 2022; Daniel et al. 2001)
	Thioridazine	CYP2D6	(Basińska-Ziobroń et al. 2025)
Sleep disorder	Modafinil	CYP2C19	(Guengerich 2022)
	Triazolam	CYP2C8	(Lai et al. 2009)
	Clopidogrel	CYP1A2, CYP2B6, CYP2C8, CYP2C9, CYP2C19	(Guengerich 2022; Lai et al. 2009; Axelsen et al. 2021; Richter et al. 2004)
Stroke	Ticlopidine	CYP1A2, CYP2B6, CYP2C19, CYP2D6, CYP2C9	(Guengerich 2022; Ko et al. 2000; Richter et al. 2004)
Viral infection	Atazanavir	CYP3A4	(Gong et al. 2019)
	Cobicistat	CYP2D6, CYP3A4	(Gong et al. 2019)
	Darunavir	CYP3A4	(Gong et al. 2019)
	Delavirdine	CYP3A4	(Guengerich 2022)
	Efavirenz	CYP1A2, CYP2C9, CYP3A4	(Guengerich 2022; Gong et al. 2019)
	Etravirine	CYP2C9, CYP2C19	(Gong et al. 2019)
	Fosamprenavir	CYP3A4	(Gong et al. 2019)
	Indinavir	CYP3A4	(Guengerich 2022; Gong et al. 2019)
	Lopinavir	CYP3A4	(Gong et al. 2019)
	Nelfinavir	CYP3A4	(Guengerich 2022; Gong et al. 2019)
	Ritonavir	CYP3A4, CYP2C8, CYP2D6	(Guengerich 2022; Lai et al. 2009; Tian and Hu 2014; Gong et al. 2019)
	Saquinavir	CYP3A4	(Guengerich 2022; Gong et al. 2019)
	Tipranavir	CYP2D6	(Gong et al. 2019)

ADHD, attention deficit hyperactivity disorder; HCV, hepatitis C virus

## Allergy

Histamine is crucial in regulating numerous physiological processes, including cell proliferation, differentiation, hematopoiesis, embryonic development, tissue regeneration, and wound healing. It acts through multiple receptor subtypes, particularly the H_1_ receptor, which rapidly affects vascular endothelial, bronchial, and smooth muscle cells. Consequently, acute manifestations such as rhinitis, bronchoconstriction, spasms, diarrhea, and skin reactions can occur (Jutel et al. 2009).

H1-antihistamines, including promethazine, chlorpheniramine, tripelennamine, diphenhydramine, and cyclizine, mitigate histamine-induced responses through H_1_ receptor blockade. These drugs inhibit CYP2D6 or CYP2C9 (He et al. 2002). An in vitro assay using human liver microsomes demonstrated strong inhibition of the bufuralol 1'-hydroxylation activity of CYP2D6 by these H1-antihistamines. Promethazine and chlorpheniramine exhibit competitive inhibition at concentrations approximating therapeutic plasma levels. In contrast, only cyclizine and promethazine inhibited tolbutamide 4-methylhydroxylation activity mediated by CYP2C9 (He et al. 2002).

CYP2B6 is essential in the hydroxylation of the antidepressant bupropion. In vitro studies demonstrate that cetirizine, chlorpheniramine, desloratadine, fexofenadine, hydroxyzine, loratadine, olopatadine, and terfenadine inhibit CYP2B6-mediated bupropion hydroxylation (Walsky et al. 2006). Moreover, loratadine and terfenadine inhibit the N-deethylation of the antimalarial drug amodiaquine, a surrogate marker reaction commonly used to assess CYP2C8 activity in vitro (Lai et al. 2009). These findings underscore the potential for DDIs between antihistamines and the metabolism of bupropion or amodiaquine.

## Asthma

CYP1A2 metabolizes caffeine into primary metabolites, including theophylline. Consequently, CYP1A2 polymorphisms can alter the metabolism and clearance of theophylline (Obase et al. 2003). Furafylline, a theophylline alternative, acts as a potent and selective inhibitor of CYP1A2 (Guo et al. 2021). Inhibition of CYP1A2 by furafylline reduces the oxidation of caffeine, leading to higher plasma caffeine levels (Wójcikowski and Daniel 2009). Similarly, concurrent administration of theophylline and furafylline suppresses the formation of theophylline metabolites, 1-methylxanthine and 1,3-dimethyl uric acid, which may affect the clinical efficacy of theophylline (Lee et al. 2014). These findings underscore the importance of considering potential DDIs in asthma therapy to optimize treatment efficacy and patient safety.

Zafirlukast, a leukotriene receptor antagonist used in asthma treatment, is primarily metabolized by CYP2C9 and CYP3A4. Consequently, genetic polymorphisms in CYP2C9 influence the biotransformation of zafirlukast (Lee et al. 2016). Zafirlukast inhibits tolbutamide 4-methylhydroxylation along with the 1-hydroxylation of midazolam, indicating its function as a CYP2C9 and CYP3A4 inhibitor, respectively (Liu et al. 2004a, b). Zafirlukast also acts as a minor inhibitor of S-mephenytoin 4'-hydroxylation catalyzed by CYP2C19, phenacetin O-deethylation (CYP1A2), and dextromethorphan O-demethylation (CYP2D6) (Liu et al. 2004a, b). Montelukast, another leukotriene receptor antagonist, serves as a potent competitive inhibitor of CYP2C9 and CYP2C8, suppressing CYP2C8-catalyzed amodiaquine N-deethylase activity (Lai et al. 2009). Pranlukast, a structural analog of zafirlukast, competitively inhibits CYP2C9 and moderately suppresses tolbutamide hydroxylation (Liu et al. 2004a, b).

## Bacterial infection

Tuberculosis, a major global public health concern and leading cause of mortality from infectious diseases, is often associated with latent infections induced by *Mycobacterium tuberculosis* in humans. Antibiotics, particularly isoniazid, are vital in preventing and treating tuberculosis (Lobue and Moser 2003). In vitro studies examining the inhibitory effect of isoniazid on CYP activity in human liver microsomes demonstrated notable inhibition of CYP1A2, 2A6, 2C9, 2C19, 2D6, 2E1, and 3A4. Specifically, isoniazid exhibits reversible inhibition of CYP2C19 and CYP3A4 activity (Wen et al. 2002). Beyond reversible inhibition, in which enzyme activity is directly impeded, CYPs can also undergo metabolic or mechanism-based inhibition. In vitro*,* isoniazid exhibits differential inhibition of CYP enzymes depending on the presence of NADPH. In the absence of NADPH, isoniazid acts as a competitive inhibitor of CYP1A2, CYP2C9, and CYP2E1; a mixed-type inhibitor of CYP2A6, CYP2C19, and CYP2D6; and an uncompetitive inhibitor of CYP3A4. However, in the presence of NADPH, isoniazid exhibits mixed-type inhibition of CYP1A2 and CYP2C19, noncompetitive inhibition of CYP2A6, and uncompetitive inhibition of CYP3A4. Therefore, the concurrent administration of isoniazid with drugs primarily metabolized by CYP2C19 and CYP3A4 may lead to significant DDIs, with potentially serious clinical implications (Wen et al. 2002; Zhou and Zhou 2009).

Erythromycin, a widely used macrolide antibiotic, acts as a mechanism-based inhibitor of CYP enzymes, with specific activity against CYP3A4 (Zhou 2008). Following erythromycin binding, the flexible active site of CYP3A4 undergoes significant conformational changes that increase its volume. Given that CYP3A4 mediates the metabolism of most drugs, the investigation of DDIs involving erythromycin and its substrates is crucial. For example, palbociclib, a CDK4/6 inhibitor metabolized by CYP3A4, exhibits significant pharmacokinetic alterations when co-administered with erythromycin. These findings underscore the importance of clinical evaluation and appropriate dose adjustments (Molenaar-Kuijsten et al. 2022).

Quinolones inhibit CYP1A2, the enzyme responsible for catalyzing caffeine 3-demethylation. Certain quinolone antibiotics containing the 4-oxoquinoline-3-carboxylic acid as a core structure competitively inhibit caffeine 3-demethylation (Zhou et al. 2010). Additionally, ciprofloxacin exhibits weak inhibitory activity against CYP1A2 and CYP2C9, while levofloxacin only inhibits CYP2C9 (Zhang et al. 2008).

## Cushing’s syndrome

Cushing’s syndrome (CS) is a rare disorder, with an annual incidence of approximately 1.8–3.2 cases per million individuals, but it can be life-threatening owing to prolonged exposure to glucocorticoids. This exposure leads to a wide range of comorbidities, including cardiovascular, metabolic, dermatological, neuropsychiatric, musculoskeletal, and reproductive symptoms (Fleseriu and Castinetti 2016; Hakami et al. 2021). Endogenous CS is classified into adrenocorticotropic hormone (ACTH)-dependent (70–80%) and ACTH-independent forms (Ma et al. 2016). Among ACTH-dependent CS cases, 80–90% arise from corticotroph pituitary adenomas (Cushing’s disease or corticotropinoma), representing approximately 65% of all CS cases, while the remaining 10–20% result from ectopic ACTH-secreting tumors (Ma et al. 2016; Gadelha et al. 2023). Transsphenoidal surgery is the first-line treatment for CS, with appropriate biochemical control of hypercortisolism to prevent disease recurrence. Medical therapy is classified into three categories: adrenal steroidogenesis inhibitors (ketoconazole, levoketoconazole, metyrapone, osilodrostat, mitotane, and etomidate), pituitary tumor-directed agents (cabergoline and pasireotide), and glucocorticoid receptor antagonists (mifepristone) (Gadelha et al. 2023). Steroidogenesis inhibitors reduce cortisol production by targeting specific enzymes: CYP11A1 (ketoconazole and etomidate), CYP11B1 (ketoconazole, levoketoconazole, metyrapone, mitotane, etomidate, and osilodrostat), CYP11B2 (ketoconazole, metyrapone, mitotane, and osilodrostat), CYP17 (ketoconazole, metyrapone, and etomidate), and CYP19 (ketoconazole, levoketoconazole, and metyrapone) (Gadelha et al. 2023; Cai et al. 2022).

## Depression

Second-generation antidepressants, particularly selective serotonin reuptake inhibitors (SSRIs), were developed to reduce the adverse effects associated with early antidepressants, including monoamine oxidase inhibitors (MAOIs) and tricyclic antidepressants (TCAs). Owing to their high selectivity for serotonin receptors, SSRIs are widely employed as monotherapy and in combination with other treatments (Ferguson 2001). SSRIs are primarily metabolized by CYPs, with single-nucleotide polymorphisms (SNPs) in CYP1A2, CYP2D6, CYP2C19, CYP2C9, and CYP3A4 significantly affecting their metabolism (Probst-Schendzielorz et al. 2015). Various antidepressants, classified according to their mechanisms of action, are metabolized by CYPs and can inhibit their activity (Table 3 & 4).

**Table 4:** Antidepressant classification based on mechanism of action and associated cytochrome P450 metabolic pathways

Antidepressant class	Mechanism of action	Drugs	Cytochrome P450 isoforms	References
MAOI	Inhibit monoamine oxidase, the enzyme that breaks down neurotransmitters, such as serotonin, norepinephrine, and dopamine	Moclobemide	CYP2C19	(Cai 2014; Borowicz-Reutt and Banach 2021)
TCA	Block the reuptake transporters for norepinephrine and serotonin	Clomipramine	CYP2C19, CYP2D6	(Mease 2009; Yokono et al. 2001; Rüdesheim et al. 2025; Kirchheiner et al. 2002; Jornil et al. 2011; Wen et al. 2008)
		Desipramine	CYP2D6	
		Doxepin	CYP2D6, CYP2C9, CYP2C19	
		Nortriptyline	CYP1A2, CYP2C19, CYP2D6, CYP3A4/5	
SSRI	Selectively inhibit serotonin reuptake	Citalopram	CYP2C19, CYP2D6, CYP3A4	(Hu et al. 2016; Mrazek et al. 2011; Owens and Rosenbaum 2002; Charlier et al. 2003; Zhang et al. 2024; Chen et al. 2020)
		Escitalopram	CYP2C19, CYP2D6, CYP3A4	
		Fluoxetine	CYP2D6	
		Fluvoxamine	CYP2D6	
		Paroxetine	CYP2D6	
		Sertraline	CYP2B6, CYP2C9, CYP2C19, CYP2D6, CYP3A4	
SNRI*	Selectively inhibit reuptake of norepinephrine and serotonin	Duloxetine	CYP1A2, CYP2D6	(Shelton 2019; Dell'osso et al. 2010; Lobo et al. 2008; Hole et al. 2024)
NDRI**	Inhibit reuptake of norepinephrine and dopamine	Bupropion	CYP2B6	(Stahl et al. 2004; Hesse et al. 2000)

^*^SNRI; serotonin-norepinephrine reuptake inhibitor **NDRI; norepinephrine dopamine reuptake inhibitor

Fluvoxamine, a selective SSRI, is a potent inhibitor of CYP1A2, which is responsible for the N3-demethylation of caffein and theophylline, and phenacetin O-deethylation (Luong et al. 2022; Ma et al. 2025a, b). Consequently, fluvoxamine may alter the metabolism of CYP1A2 substrate drugs (Britz et al. 2019).

Most antidepressants are metabolized primarily by CYP2D6 (Haufroid and Hantson 2015). Paroxetine, a substrate of this enzyme, acts as a potent inhibitor of CYP2D6, hindering the metabolic clearance of desipramine, a tricyclic antidepressant generated from imipramine via CYP2C19. Consequently, co-administration of desipramine with paroxetine necessitates a substantial dosage reduction (Zakaraya et al. 2024). Furthermore, bupropion—a CYP2B6 substrate—and its metabolite hydroxybupropion inhibit CYP2D6, thereby reducing desipramine metabolism. Hence, caution is required when administering desipramine (Kotlyar et al. 2005).

## Epilepsy

**Fig. 3:**
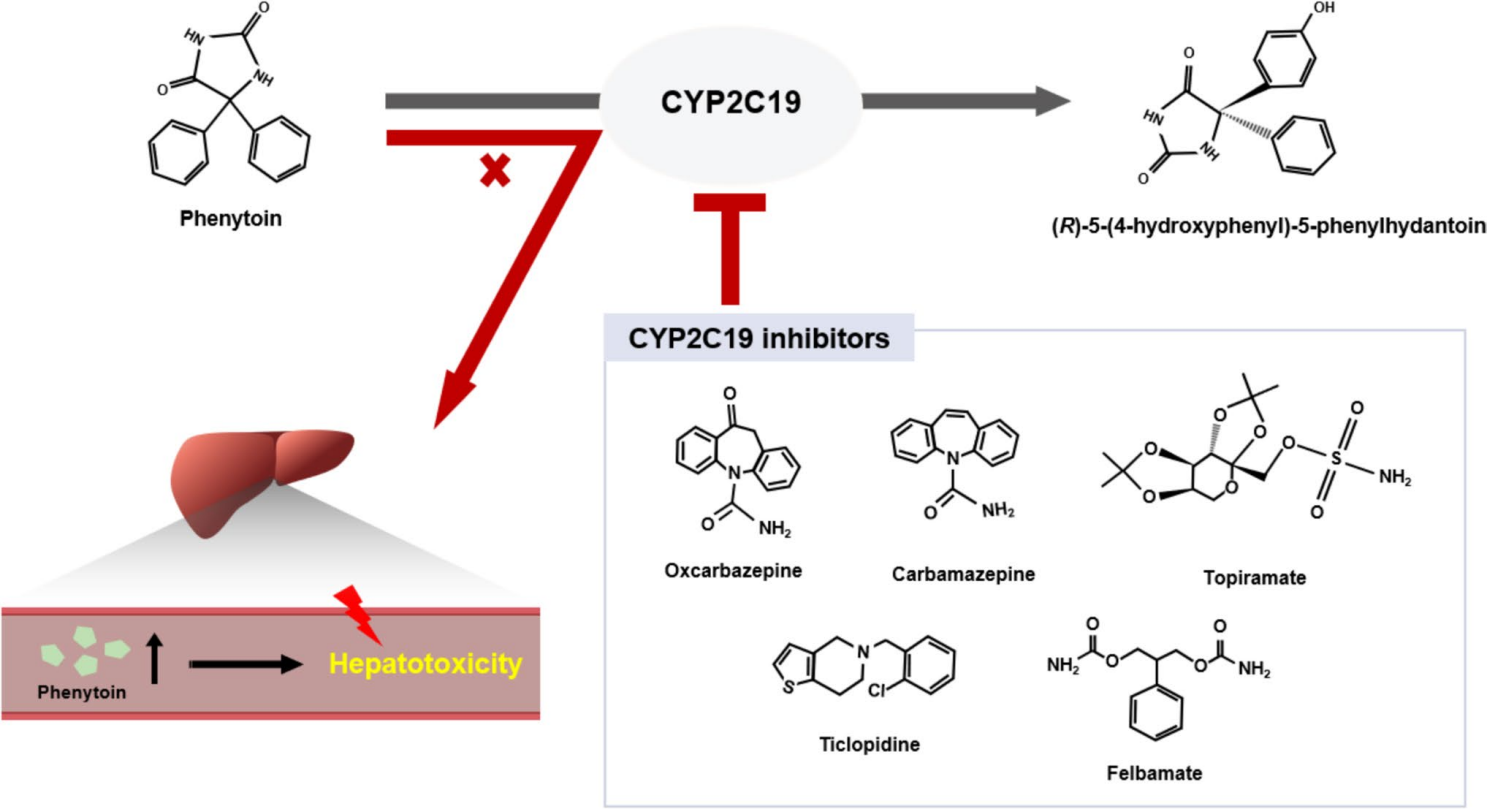
Inhibition of phenytoin metabolism by CYP2C19 inhibitors and the resulting hepatotoxicity. Phenytoin is metabolized in the liver by CYP2C19 into the major metabolite 5-(4-hydroxyphenyl)-5-phenylhydantoin. Several antiepileptic agents, such as oxcarbazepine, carbamazepine, topiramate, ticlopidine, and felbamate, inhibit this pathway (Flaten et al. 2016; Prentice et al. 2022; Malavé et al. 2025). Co-administration of these CYP2C19 inhibitors with phenytoin reduces its metabolism, leading to metabolite formation. This alteration in phenytoin pharmacokinetics increases plasma drug concentrations and elevates the risk of phenytoin-induced hepatotoxicity

Antiepileptic drugs metabolized by hepatic CYP enzymes are influenced by CYP inhibitors, potentially resulting in metabolic disruption and subsequent adverse effects. For example, valproic acid is primarily metabolized by CYP2C9 and CYP2C19; thus, alterations in their activities can elevate plasma valproic acid concentrations (Song et al. 2022a, b). Similarly, phenytoin is metabolized predominantly by CYP2C9 (90%) and to a lesser extent by CYP2C19 (10%) (Rodriguez-Vera et al. 2023). Additionally, CYP2C19 activity can be inhibited by other antiepileptic drugs such as oxcarbazepine, topiramate, and carbamazepine (Lakehal et al. 2002; Bialer et al. 2004). These CYP2C9 inhibitors interfere with the formation of the primary phenytoin metabolite, (R)-5-(4-hydroxyphenyl)-5-phenylhydantoin (Coelho et al. 2021) (Fig. 3). Clinically, co-administering CYP2C9/CYP2C19 inhibitors with phenytoin elevates plasma phenytoin concentrations, which may trigger severe hepatic injury induced through hypersensitive reactions resembling immunoallergic hepatotoxicity (Soskin et al. 2010; Tabrizi and Sharifi-Razavi 2022; Bhavsar et al. 2023) (Fig. 3). Therefore, potential DDIs among antiepileptic drugs should be considered when designing dosing regimens to prevent hepatotoxicity.

## Gout

Lesinurad, a uricosuric drug used in the treatment of gout, is primarily metabolized by CYP2C9. Genetic polymorphisms in CYP2C9 can increase plasma concentrations of lesinurad. Therefore, caution is warranted when co-administering CYP2C9 inhibitors with lesinurad, as they may affect its metabolism and further elevate plasma drug levels (Dean 2012). Lesinurad may act as a weak inhibitor of CYP2C9 (Shen et al. 2019). However, this inhibitory effect is negligible, allowing the substrates of the CYP2C9 enzyme to be significantly affected during metabolic processes by benzbromarone, a potent inhibitor of CYP2C9 (Locuson et al. 2003).

Benzbromarone, a uricosuric agent, inhibits CYP2C9 and CYP3A4. In particular, it structurally inactivates CYP3A4 through irreversible covalent adduction. This can result in clinically significant DDIs and is linked to benzbromarone-induced fatal hepatotoxicity (Masubuchi and Kondo 2016; Tang et al. 2021).

## Hyperlipidemia

In premenopausal women, estrogen plays a key role in increasing high-density lipoprotein levels while lowering low-density lipoprotein (LDL) and triglyceride levels, thereby reducing the risk of hyperlipidemia compared with men of the same age. CYP2C9 contributes to the metabolism of estrogens, including estradiol, estrone, and progesterone. Consequently, genetic polymorphisms in CYP2C9 may alter estrogen concentrations and contribute to the development of hyperlipidemia (Luo et al. 2005). Gemfibrozil, which is used to treat hyperlipidemia by lowering LDL and triglyceride levels, is a potent inhibitor of CYP2C8 and CYP2C9 (Wen et al. 2001; Davidson 2006). In vitro, gemfibrozil only weakly inhibits CYP2C8. However, once glucuronidated to form its metabolite, gemfibrozil 1-O-β-glucuronide, it becomes a significant CYP2C8 inhibitor in vivo (Tornio et al. 2017).

A study on using pooled human liver microsomes demonstrates that gemfibrozil is a potent, competitive inhibitor of CYP2C9. This suggests that gemfibrozil may interact with CYP2C9 substrate drugs such as warfarin or glyburide (Wen et al. 2001). Additionally, co-administration of cerivastatin, a cholesterol-lowering statin, with gemfibrozil, a CYP2C8 inhibitor, interferes with the production of the hydroxy metabolite of cerivastatin. These findings highlight the importance of considering potential DDIs when prescribing gemfibrozil with other CYP2C8-metabolized medications (Wang et al. 2002).

## Hypertension

CYP enzymes metabolize arachidonic acid in the brain and blood vessels to generate metabolites such as EETs, dihydroxyeicosatrienoic acids (DiHETEs), and HETEs. These metabolites are crucial in cardiovascular diseases, including hypertension (Sarkis and Roman 2004). Meanwhile, increasing research efforts have focused on investigating CYP inhibition by existing antihypertensive drugs.

Diltiazem, a calcium channel blocker widely prescribed for hypertension and angina, is primarily metabolized by CYP3A4 (Zisaki et al. 2015). Diltiazem is a moderate CYP3A4 inhibitor, but its metabolites, N-desmethyldiltiazem and N,N-didesmethyldiltiazem, are very strong reversible inhibitors of CYP3A. Consequently, prolonged exposure to diltiazem in humans causes these metabolites to accumulate in the blood, which further inhibits CYP3A4 and reduces diltiazem clearance (Zhao 2008; Byeon et al. 2018). Given the central role of CYP3A4 in metabolizing numerous drugs, characterizing interactions between diltiazem and other CYP3A4 substrates is of considerable interest. For example, in a study involving 10 healthy volunteers, co-administering simvastatin—a cholesterol-lowering drug metabolized by CYP3A4—and diltiazem resulted in a significant interaction, underscoring the need for caution when prescribing simvastatin with diltiazem or other CYP3A4 inhibitors (Mousa et al. 2000).

## Malaria

Antimalarial drugs, including hydroxychloroquine, are primarily metabolized in the liver by CYP2D6, CYP3A4, and CYP2C8 (Rendic and Guengerich 2020). Genetic polymorphisms in these enzymes can significantly influence drug metabolism (Elewa and Wilby 2017). Chloroquine has been reported to suppress CYP2D6 activity, potentially through autoinhibition of its own metabolism. Consistently, hydroxychloroquine, its less toxic derivative, has also been shown to significantly inhibit this enzyme, suggesting that both agents may impact the metabolism of CYP2D6 substrates (Chamboko et al. 2023; Paludetto et al. 2023). When co-administering CYP2D6 inhibitors with other therapies to treat malaria or other inflammatory diseases, evaluating potential DDIs is essential, as they may affect the metabolism of CYP2D6 substrates (Rendic and Guengerich 2020).

## Mycosis

The antifungal drugs ketoconazole, fluconazole, voriconazole, and itraconazole are potent inhibitors of CYP3A4 (Guengerich 2022), as demonstrated in studies of testosterone metabolism. Ketoconazole, voriconazole, and fluconazole exhibit comparable inhibitory effects, while itraconazole produces distinct effects, potentially due to its larger molecular size, which enables tighter binding to the CYP3A4 catalytic site (Yamaguchi et al. 2021). Ketoconazole is a strong CYP3A inhibitor that effectively hinders the biotransformation of CYP3A substrates (Greenblatt et al. 2011; Yamaguchi et al. 2021), with its inhibitory potency varying in a substrate-dependent manner (Greenblatt et al. 2011). Fluconazole also inhibits CYP2C9 and CYP2C19, resulting in DDIs that enhance the anticoagulant effects of warfarin (Akamatsu et al. 2023).

The antifungal agent terbinafine acts as a CYP2D6 inhibitor and induces a discrepancy between the genotype and phenotype of the enzyme. Consequently, concomitant administration of terbinafine may significantly influence the biotransformation of CYP2D6 substrates (Nahid et al. 2025).

## Peptic ulcers

Polymorphisms in CYP2C19 are associated with peptic ulcer disease (Sychev et al. 2015). The distribution of CYP2C19 genotypes varies among patients with gastrointestinal disease, and rapid metabolizer genotypes may increase susceptibility to peptic ulcer disease and gastrointestinal bleeding (Jainan and Vilaichone 2014). *Helicobacter pylori* (*H. pylori*) infection significantly increases the risk of peptic ulcers, gastritis, and gastric cancer (Liou et al. 2024). Therefore, eradicating *H. pylori* is a key component of treatment strategies for patients with peptic ulcer disease (Kurzawski et al. 2006). Proton pump inhibitors (PPIs) are commonly used in *H. pylori* eradication therapy, with CYP2C19 playing a crucial role in their metabolic processing. Thus, PPI metabolism is influenced by CYP2C19 polymorphisms, which influence treatment outcomes in patients with peptic ulcer disease (Klotz et al. 2004; Kurzawski et al. 2006).

Several PPIs that serve as CYP2C19 substrates also function as inhibitors of the same enzyme. For example, esomeprazole and omeprazole are clinically potent inhibitors of CYP2C19 (Zvyaga et al. 2012). In vitro, esomeprazole shows time-dependent inhibition of CYP2C19, with higher doses producing stronger inhibitory effects (Kaartinen et al. 2020). Additionally, omeprazole and its metabolites reversibly inhibit CYP2C19, and DDIs involving other CYP2C19 substrates are well documented (Malling et al. 2005).

Cimetidine is the first FDA-approved histamine H_2_-receptor antagonist that effectively inhibits gastric acid secretion (Khawaja et al. 2024). It is also a well-recognized inhibitor of CYP2C19 and other CYP enzymes, including CYP1A2, CYP2C9, CYP2D6, and CYP3A4/5 (Malling et al. 2005; Guengerich 2022). Patients undergoing treatment with drugs metabolized and deactivated by CYP3A4 may experience enhanced drug effects due to pharmacokinetic interactions when co-administered with cimetidine, a competitive inhibitor for CYP3A4 (Wendl et al. 2022).

## Psoriasis

Psoriasis is recognized as a chronic autoimmune skin condition accompanied by systemic inflammation, exerting wide-ranging effects that extend beyond the skin and influence cardiometabolic, renal, malignant, and psychological health (Bu et al. 2022). CYPs play significant roles in the pathogenesis of various skin diseases (Chen et al. 2024a, b). CYP2S1 may contribute to psoriasis development by inhibiting keratinocyte proliferation and regulating immune response pathways (Sheng et al. 2021).

Methoxsalen, a natural coumarin which is a well-known medication for psoriasis, functions as a potent therapeutic agent for lung cancer through selective inhibition of CYP2A6 and CYP2A13 (Sharma et al. 2024). CYP2A6 metabolizes nicotine into cotinine, thereby influencing smoking behaviors and lung cancer risk. Methoxsalen can potentially reduce smoking behavior in vivo by inhibiting CYP2A6-mediated nicotine metabolism (Zhang et al. 2001; Zhu et al. 2013). Furthermore, methoxsalen is a potent inhibitor of CYP1A2, markedly reducing phenacetin *O*-deethylation activity (Palacharla et al. 2019).

## Schizophrenia

The metabolism of most antipsychotic medications depends on various CYP isoforms, rendering them susceptible to alterations when subjected to antipsychotics capable of concurrently inhibiting CYPs (Basińska-Ziobroń et al. 2025). Olanzapine, commonly prescribed for schizophrenia and related conditions, is primarily metabolized by CYP1A2. Consequently, fluvoxamine—a CYP1A2 inhibitor—significantly increases the peak plasma concentrations and reduces the clearance of clozapine and olanzapine, indicating that lower doses of the second-generation antipsychotics may be required to maintain therapeutic levels when co-administered with fluvoxamine (Lenze et al. 2022; Mahgoub et al. 2023).

Several antipsychotic drugs––including chlorpromazine, fluphenazine, haloperidol, perphenazine, and thioridazine––have been identified as CYP2D6 inhibitors (DeBattista and Schatzberg 2024; Basińska-Ziobroń et al. 2025). Studies assessing the inhibition of bufuralol 1′-hydroxylation and codeine *O*-demethylation, a CYP2D6-mediated reaction, demonstrate competitive inhibition (Vevelstad et al. 2009; Wójcikowski et al. 2020). Among these, thioridazine and perphenazine exhibited the greatest potency with *K*_*i*_ values of 1.4 and 0.8 μM, respectively (Basińska-Ziobroń et al. 2025). Moreover, fluphenazine and perphenazine moderately inhibit CYP1A2-mediated phenacetin *O*-deethylation (Daniel et al. 2001).

## Stroke

When CYP enzymes metabolize arachidonic acid, they produce eicosanoids that regulate cerebral blood flow. CYP-mediated eicosanoid-induced cerebrovascular dysfunction plays a significant role in stroke development and progression (Huang et al. 2016). Additionally, polymorphisms in the CYP promoter increase ischemic stroke risk. A clinical trial involving 121 patients with ischemic stroke reports associations between nucleotide polymorphisms in CYP11B2, CYP2E1, and CYP7A1 and the occurrence of ischemic stroke (Kim et al. 2012). Another trial involving patients with ischemic stroke and carotid stenosis shows that CYP polymorphisms alter plasma levels of CYP-derived metabolites, including EETs, DiHETEs, and HETEs, which increase the risk of carotid stenosis (Yi et al. 2016). These findings highlight the importance of considering CYP inhibition as a potential strategy in stroke treatment.

Ticlopidine, an antiplatelet drug commonly used in managing thrombotic stroke, inhibits CYPs. In vitro studies using human liver microsomes report ticlopidine as a potent competitive inhibitor of CYP2C19 and CYP2D6. It also inhibits CYP1A2 and CYP2C9 (Ko et al. 2000). Clopidogrel, another thienopyridine antiplatelet agent, inhibits CYP2C8, with significant implications for studies evaluating the effects of co-administering CYP2C8 substrates (Axelsen et al. 2021). Additionally, ticlopidine and clopidogrel exhibit mechanism-based inhibition of CYP2B6, characterized by time- and concentration-dependent, irreversible NADPH-dependent inhibition (Richter et al. 2004).

## Viral infection

Several antiretroviral drugs used for managing HIV/AIDS are CYP inhibitors (Gong et al. 2019). These drugs are often administered in combination therapy to target different stages of the HIV life cycle and slow disease progression (De Clercq 2009). Ritonavir and cobicistat, potent CYP3A4 inhibitors, mitigate the need for higher doses and frequent administration of CYP3A4-metabolized antiretroviral drugs (Gong et al. 2019). Furthermore, ritonavir has more recently been repurposed to treat COVID-19 (Meini et al. 2020). Paxlovid, a combination of ritonavir and nirmatrelvir, was the first FDA-approved oral treatment for COVID-19 granted Emergency Use Authorization (Saheb Sharif-Askari et al. 2024). Nirmatrelvir inhibits the 3-chymotrypsin-like cysteine protease of SARS-CoV-2, while ritonavir enhances its antiviral effect by interfering with CYP3A4-induced metabolism of nirmatrelvir (Amani and Amani 2023). Clinical inhibition of CYP3A4 by ritonavir is characterized according to its nearly irreversible nature, requiring new enzyme synthesis for functional recovery (Loos et al. 2022). Additionally, ritonavir inhibits CYP2C8 and CYP2D6 (Lai et al. 2009; Guengerich 2022).

Antiretroviral medications exhibit complex interactions with CYP metabolic pathways. For instance, efavirenz is primarily metabolized by CYP2B6 and concurrently inhibits CYP1A2, CYP2C9, and CYP3A4. Similarly, etravirine is metabolized by CYP3A4, CYP2C9, and CYP2C19, while also inhibiting CYP2C9 and CYP2C19 (Stolbach et al. 2015; Gong et al. 2019). Moreover, retroviral protease inhibitors, such as atazanavir, darunavir, fosamprenavir, lopinavir, saquinavir, indinavir, and nelfinavir, generally exert intermediate to weak inhibitory effects on CYP3A4. Conversely, tipranavir specifically inhibits CYP2D6 (Gong et al. 2019).

## Future perspectives

Recognizing the significance of disease-causing CYP metabolites has driven active research into developing drugs that target and inhibit CYP enzymes. Earlier studies primarily focused on evaluating the potency and mechanisms of action of existing CYP inhibitors. More recently, advances in structural analyses have offered comprehensive insights into why several current CYP inhibitors lack selectivity. This knowledge supports the rational design of highly selective CYP inhibitors with potential for targeted therapeutic interventions (Zhao et al. 2019). For example, studies show the efficacy of CYP1A inhibitors in cell-based assays, suggesting potential for developing selective inhibitors for other CYP isoforms (Dai et al. 2024).

Despite this progress, various CYP inhibitors remain underexplored, while known inhibitors may possess additional functions with the potential to transform disease treatment. Ongoing research into the structural properties and mechanisms of CYP inhibitors has led to the development of novel drugs with promise for groundbreaking treatments and diverse therapeutic strategies. Furthermore, combining these inhibitors with other medications requires careful assessment of DDIs (Table 5) to optimize therapeutic efficacy and minimize adverse effects. A comprehensive understanding of these interactions is crucial for effectively integrating CYP inhibitors into clinical practice.

**Table 5:** Examples of CYP inhibitor-derived DDIs

Target drug for DDI	Function of target drug	CYP enzymes as metabolizers	CYP inhibitor drugs for DDI	Related disease
Amodiaquine Bupropion Chloroquine Cyclosporin Erlotinib	Antimalarial agent Antidepressant Antimalarial agent Immunosuppressive drug First-generation TKI targeting EGFR	CYP2C8 CYP2B6 CYP2D6 CYP3A4 CYP3A4	Loratadine, terfenadine Cetirizine, chlorpheniramine, Desloratadine, fexofenadine, Hydroxyzine, loratadine, olopatadine, terfenadine Halofantrine Imatinib Ketoconazole, clarithromycin, voriconazole	Allergy (antihistamine drugs) Allergy (antihistamine drugs) Malaria, inflammatory disease CML Advanced or metastatic NSCLC
Glyburide Nirmatrelvir Palbociclib PPIs Statins Theophylline Valproic acid/phenytoin Warfarin	Antidiabetic medication Antiviral SARS-CoV-2 protease inhibitor CDK4/6 inhibitor HMG-CoA reductase inhibitors for hypercholesteremia Asthma therapy Anticonvulsant drugs for epilepsy Anticoagulant agent	CYP2C9 CYP3A4 CYP3A4 CYP2C19 CYP2C8 CYP1A2 CYP2C19 CYP3A4, CYP2C9	Gemfibrozil Ritonavir Erythromycin Esomeprazole, omeprazole gemfibrozil Furafylline Oxcarbazepine, topiramate, carbamazepine Fluconazole, gemfibrozil	Type 2 diabetes COVID-19 ER-positive metastatic breast cancer (postmenopausal) Peptic ulcer Hyperlipidemia Asthma Epilepsy Mycosis, hyperlipidemia

TKI, tyrosine kinase inhibitor; EGFR, epidermal growth factor receptor; CML, chronic myeloid leukemia; NSCLC, non-small cell lung cancer; SARS-CoV-2, severe acute respiratory syndrome coronavirus 2; PPI, proton pump inhibitor; HMG-CoA, 3-hydroxy-3-methylglutaryl-coenzyme A

## Data Availability

All data needed to evaluate the conclusions in the paper are present in the paper.
